# lncRNA *PVT1* Promotes Metastasis of Non-Small Cell Lung Cancer Through EZH2-Mediated Activation of Hippo/NOTCH1 Signaling Pathways

**DOI:** 10.22074/cellj.2021.7010

**Published:** 2021-03-01

**Authors:** Shang-Gan Zeng, Jian-Hong Xie, Qun-Ying Zeng, Shao-Hua Dai, Yun Wang, Xue-Mei Wan, Ji-Chun Liu

**Affiliations:** 1. Department of Thoracic Surgery, The First Affiliated Hospital of Nanchang University, Nanchang 330006, P.R China; 2. Department of Surgery, Suichuan People’s Hospital, Ji'an 343900, P.R China; 3. Department of Cardiovascular Surgery, The First Affiliated Hospital of Nanchang University, Nanchang, 330006, P.R China

**Keywords:** EZH2, miR-497, NSCLC, *PVT1*, YAP1

## Abstract

**Objective:**

Although growing evidences have showed that long non-coding RNA (lncRNAs) plasmacytoma variant translocation 1 (*PVT1*) plays a critical role in the progression of non-small cell lung cancer (NSCLC), there are still many unsolved mysteries remains to be deeply elucidated. This study aimed to find a new underlying mechanism of *PVT1* in regulating the tumorigenesis and development of NSCLC.

**Materials and Methods:**

In this experimental study, Quantitative reverse transcription polymerase chain reaction (qRTPCR) was used to profile the expression of *PVT1* in NSCLC tissues and cells. The effects of *PVT1* on cell growth, migration and invasion were detected by colony formation assay, Matrigel-free transwell and Matrigel transwell assays, respectively. Changes of the key protein expression in Hippo and NOTCH signaling pathways, as well as epithelialmesenchymal transition (EMT) markers, were analyzed using western blot. Interaction of *PVT1* with enhancer of zeste homolog 2 (EZH2) was verified by RNA pull-down, and their binding to the downstream targets was detected by Chromatin Immunoprecipitation (ChIP) assays.

**Results:**

These results showed that *PVT1* was up-regulated in NSCLC tissue and cell lines, promoting NSCLC cell proliferation, migration and invasion. Knockdown of *PVT1* inhibited the expression of Yes-associated protein 1 (YAP1) and NOTCH1 signaling activation. Further, we have confirmed that *PVT1* regulated expression of YAP1 through EZH2-mediated miR-497 promoter methylation resulting in the inhibition of miR-497 transcription and its target YAP1 upregulation, and finally NOTCH signaling pathway was activated, which promoted EMT and invasion and metastasis.

**Conclusion:**

These results suggested that lncRNA *PVT1* promotes NSCLC metastasis through EZH2-mediated activation of Hippo/NOTCH1 signaling pathways. This study provides a new opportunity to advance our understanding in the potential mechanism of NSCLC development.

## Introduction

Lung cancer is the leading cause of cancer-related death worldwide (1). The most common type of that is non-small cell lung cancer (NSCLC), which accounts for approximately 85% of all lung cancer new cases (2). The average 5-year survival rate of NSCLC cancer patients is still very low, because of the limited therapeutic options, I addition to the higher rate of tumor metastasis and recurrence (2).

Yes-associated protein 1 (YAP1) is highly expressed in NSCLC tissues and cells. It can positively regulate expression of NOTCH1, affecting proliferation, invasion and metastasis ability as well as drug sensitivity in lung cancer cells (3). Our previous work proved that YAP, a core transcription co-activator in Hippo signaling pathway, was overexpressed in NSCLC tissues and cells, positively regulated expression of NOTCH1 and markedly promoted cell proliferation and invasion (4). These results indicated that Hippo and NOTCH signaling pathways played an important role in development of NSCLC. However, the specific molecular mechanisms of these two signaling pathways in NSCLC tumorigenesis and development are not fully understood yet.

Long non-coding RNAs (lncRNAs) are non-coding transcripts with longer than 200 nucleotides, which exhibit various functions and regulate different processes by many molecular mechanisms (5). Growing evidences suggest that lncRNAs participate in the development and progression of NSCLC.* MALAT1* was reported to be a predictive marker for NSCLC metastasis development (6), while elevated expression of LINC00473 correlated with poor prognosis of NSCLC (7). Plasmacytoma variant translocation 1 (*PVT1*), a lncRNA that shares the location of chr8q24.21 with *c-Myc* (8). It is highly expressed and exerts a carcinogenic effect in many tumors, such as NSCLC (9), colorectal cancer (10) and hepatocellular carcinoma (11). Recently, it was reported that *PVT1* recruited enhancer of zeste homolog 2 (EZH2) to the large tumor suppressor kinase 2 (*LATS2*) promoter and repressed its transcription (9). Therefore, *PVT1* knockdown could inhibit proliferation and induce apoptosis in NSCLC (9).

LATS2 plays a pivotal role in regulating Hippo growth inhibitory signaling (12). Recent study showed that LATS2 inhibition decreased YAP1 phosphorylation. It promoted nuclear accumulation of YAP1 and upregulated the association of YAP1/ TEA domain transcription factor 2 (TEAD2), which led to transcriptional activation of YAP1/TEAD2 (12). These results indicated that lncRNA *PVT1* may inhibit Hippo signaling by silencing LATS2, and it plays a crucial role in promoting proliferation and anti-apoptosis. It was also reported that knockdown of lncRNA *PVT1* inhibited cell viability, invasion and induced apoptosis in NSCLC by regulating *miR-497* expression (13). However, the mechanism by which lncRNA *PVT1* inhibits *miR-497* still needs to be elucidated.

NOTCH signaling was reported to be altered in approximately one third of NSCLCs (14). Numerous studies have also suggested that activation of NOTCH correlates with poor clinical outcomes in NSCLC patients without *TP53* mutations and it is a biomarker for predicting survival time in patients with NSCLC (15). However, mechanism of NOTCH1 up-regulation is not well understood (14). An interacting network of the Hippo and NOTCH signaling pathways that control organ size and hepatocellular carcinoma (HCC) development was also identified (16). NOTCH and Hippo signaling was also showed to synergize to potentiate liver cell growth and remodel (17). Jagged-1 and NOTCH2, two NOTCH pathway components, are downstream targets of Hippo signaling. They lead to the dedifferentiation of hepatocytes into hepatic progenitors (18). Besides, YAP-dependent activity of Jag1 and Notch were also reported to correlate with survival times in human HCC and colorectal tumor samples (19). YAP1 can also contribute to progression and poor prognosis of NSCLC (4). Thus, we hypothesized that under the mediation of TEAD1, YAP1 is most likely a NOTCH1 upstream driver gene. We proposed hypotheses that lncRNA *PVT1* interacts with EZH2 to silence the expression of *miR-497* and LATS2 genes. Thus, it promotes YAP1 transcription and inhibits phosphorylation of YAP1, thereby activating NOTCH1 signaling and enhancing the invasion of NSCLC cells.

## Materials and Methods

### Tissue collection

This experimental study was approved by the Ethics Committee (Code No.: 20180521) of the First Affiliated Hospital of Nanchang University (Nanchang, China). Written informed consents were obtained from all patients. Thirty paired primary tumor tissues and adjacent tissues from these NSCLC patients were obtained. Clinical-pathological characteristics were recorded. No local or systemic treatment was conducted in these patients before surgery. All samples were immediately snap-frozen in liquid nitrogen and stored at -80˚C, until required. 

### Cell lines and cell culture

Human NSCLC cell lines A549, H1299, Calu-3, H1975 and PC-9 as well as human bronchial epithelial cells BEAS-2B were obtained from the American Type Culture Collection (ATCC, USA), cultured in their corresponding medium containing 10% FBS (Gibco, USA), 100 μg/ml streptomycin (HyClone, USA) and 100 U/ml penicillin (HyClone) and incubated at 37˚C in the presence of 5% CO2 .

### RNA extraction and quantitative reverse transcription PCR

Trizol regent (Invitrogen, USA) was used to extract total RNA from tissue specimens and cell samples. First-strand cDNA was generated by ImProm-II Reverse Transcription System (Promega, USA). Then, SYBR Green qPCR assay (Takara, Japan) and gene-specific primers (Table 1) were used for quantitative reverse transcription PCR (qRT-PCR) analysis. *GAPDH *or *U6* was used as internal references for normalization. The relative expression levels of target genes were calculated using the comparative Ct method.

### Plasmid generation and cell transfection 

The *PVT1* sequence was synthesized and sub-cloned into the pcDNA3.1 vector (Invitrogen, USA) by GenePharma (Shanghai, China). The siRNAs directly against human 

*PVT1* gene (si-PVT1-1:

5´-CCTGTTACACCTGGGATTT-3´;

si-PVT1-2: 5´-GGACTTGAGAACTGTCCTT-3´; 

si-PVT1-3: 5´-CCTGGGATTTAGGCACTTT-3´), 

*EZH2* gene (si-EZH2: 

5´-CATCGAAAGAGAAATGGAATT-3´), 

YAP1 gene (si-YAP1: 

5´-AGAACTGCTTCGGCAGGAG-3´) 

were also designed and synthesized by GenePharma (Shanghai, China). si-NC (5´-UUCUCCGAACGUGUCACGUTT-3´) was used as a negative control. Plasmid vectors and siRNA oligonucleotides were transfected into H1299 or A549 cells with Lipofectamine 3000 (Invitrogen, USA) according to the manufacturer’s instructions. Forty-eight hours after transfection, the cells were harvested for qRT-PCR or western blot analysis

**Table 1:** Paired primer sequences used in qRT-PCR


Genes	Paired primers	Sequences (5´-3´)

*PVT1*	sense	CTTGCGGAAAGGATGTTGGC
	antisense	GCCATCTTGAGGGGCATCTT
*YAP1*	sense	TTCGGCAGGCAATACGGAAT
	antisense	GTTGAGGAAGTCGTCTGGGG
*TEAD1*	sense	CCCTGGCTATCTATCCACCA
	antisense	AGGGCCTTATCCTTTGCAGT
*NOTCH1*	sense	GCACGTGTATTGACGACGTTG
	antisense	GCAGACACAGGAGAAGCTCTC
*LATS2*	sense	ACAAGATGGGCTTCATCCAC
	antisense	CTCCATGCTGTCCTGTCTGA
*EZH2*	sense	AAGCACAGTGCAACACCAAG
	antisense	CAGATGGTGCCAGCAATAGA
*GAPDH*	sense	CCAGGTGGTCTCCTCTGA
	antisense	GCTGTAGCCAAATCGTTGT
*miR-497*	RT	GTCGTATCCAGTGCAGGGTCCGAGGTATTCGCACTGGATACGACACAAAC
	sense	GCGCAGCAGCACACTGTG
	antisense	GTGCAGGGTCCGAGGT
*U6*	sense	CTCGCTTCGGCAGCACA
	antisense	AACGCTTCACGAATTTGCGT


### Methylation-specific PCR

Methylation analysis of miR-497 promotor was examined by Methylation-specific PCR (MSP). MethPrimer 1.0 was used to design MSP primers. A pair of methylation-specific primers (M-F: 5´-TTTGATTTAGGGAGAGGAAGGAC-3´; M-R: 5´-TAAACAAACAACTAAAAAACGACGA-3´) and a pair of unmethylation-specific primers at the same site (UF: 5´-TTTGATTTAGGGAGAGGAAGGAT-3´; M-R: 5´-TAAACAAACAACTAAAAAACAACAAA-3´) were chosen. Briefly, the isolated genomic DNA was treated with sodium bisulfite using an EZ DNA Methylation Gold kit (Zymo Research, USA). They were then subjected to PCR assay using the specific primers. The PCR products were digested with a restriction endonuclease BstUI, recognizing sequences unique to the methylated alleles, but not unmethylated alleles. The digested products were next electrophoresed on 3% agarose gels and stained with ethidium bromide. The ratio of gray scale value of the methylated band was calculated as methylation levels.

### *In vitro* transcription and RNA pull-down assay

*In vitro* transcription and RNA pull-down assay were performed as described before (20). Briefly, biotin-labeled lncRNA *PVT1* was transcribed with T7 RNA polymerase by TranscriptAid T7 High Yield Transcription Kit (Thermo Fisher Scientific, USA) *in vitro*. For RNA pulldown assay, 5 μg of biotin-labeled synthesized RNA was added to the RNA structure buffer (10 mM Tris pH=7, 0.1 M KCl, 10 mM MgCl2) to ensure the formation of proper secondary structure. Following the indicated treatment, the cell samples were collected and their extracts were then mixed with biotin-labeled RNA. They were next rotated at room temperature for one hour, and then 50 μl of streptavidin-agarose beads were added to the mixture and rotated for one hour. After incubation, the beads were washed briefly twice with high-salt RNA Binding Protein Immunoprecipitation (RIP) buffer (containing 500 mM KCl, 25 mM Tris pH=7.4, 0.5 mM DTT, 0.5% NP40, 1 mM PMSF and protease inhibitor), then twice with lowsalt RIP buffer (composed of 150 mM KCl, 25 mM Tris pH 7.4, 0.5 mM DTT, 0.5% NP40, 1 mM PMSF and protease inhibitor), and lastly boiled in SDS-loading buffer for 10 minutes. The retrieved proteins were detected by means of western blotting.

### Colony formation assay

Cells after transfection were collected at logarithmic growth phase. Then, they were placed in a 6-well plate (1×10^3^ /well) for two weeks. 4% paraformaldehyde was used to fix the cells for 15 minutes after discarding the medium, and Giemsa solution was added to stain for 5 minutes. The cells were then quantified by photographing three independent visual fields under the microscope.

### Chromatin immunoprecipitation assays

ChIP assays were conducted using the SimpleChIP® Plus Enzymatic Chromatin IP Kit, according to the manufacturer’s instructions (CST, USA). H3 trimethyl Lys 27 antibody was obtained from Millipore (USA). EZH2 (5246) antibody was obtained from CST. Quantification of immunoprecipitated DNA was performed by quantitative PCR (qPCR). ChIP data were calculated as a percentage relative to the input DNA.

### Transwell migration assay

8 mm pore 24-well transwell chambers (Corning, USA) were used for migration assay. 2×10^4^ A549 or H1299 cells were seeded into the chambers and cultured with DMEM for 48 hours. Then, took out membranes at the bottom of chambers, and removed the cells on the upper membrane surface using a cotton swab. The cells on the lower surface of membrane surface were fixed with methanol and glacial acetic acid, at the ratio of (3:1) and they were stained with 10% Giemsa solution. Finally, five fields were selected randomly and counted for statistical analysis in each groups.

### *In vitro* Matrigel invasion assay

Before seeding cells, the poly-carbonate membranes of the transwell upper chambers (8 µm pore size; Corning, USA) was pre-coated with Matrigel (BD, USA). Then, 4×10^5^ cells, re-suspended in 200 µl serum-free medium, were placed in the upper chamber, followed by adding 600 µl of the same medium to the lower chamber. Then, the cells on the upper membrane surface were removed after 48 hours incubation at 37˚C. Meanwhile, the cells on the lower membrane surface were fixed with methanol and glacial acetic acid (3:1). They were next stained with 10% Giemsa solution. Finally, five fields selected randomly and counted for statistical analysis in each groups.

### Western blot analysis

The cells were harvested and protein was isolated by IP lysis buffer (Thermo Fisher Scientific, USA) containing protease inhibitors (Roche, Switzerland). Then, the BCA Assay Kit (Thermo Fisher Scientific) was used to assess the concentration of proteins in the supernatants of cell lysates. Next, 10% SDS-PAGE gel electrophoresis was applied for separation of equal amount of protein samples. Then, they were transferred to PVDF membranes, which was later incubated with a specific primary antibody followed by incubating with secondary antibody marked by horseradish peroxidase (goat anti-rabbit; Abcam) at room temperature for one hour. Optical density method was used for quantitative autoradiography with β-actin (1:3000; Proteintech, USA), as controls. 

### Statistical analysis

Prism 6.0 (GraphPad Software, USA) was used for statistical analysis of data. All experiments were performed at least three times in triplicate. Data were expressed as the mean ± standard deviation (SD). Student’s t test (two tailed) and one-way analysis of variance (ANOVA) were used to evaluate the significant difference. P<0.05 was considered to be significantly different.

## Results

### lncRNA *PVT1* was upregulated in NSCLC tissues and cell lines, promoting cell proliferation, migration and invasion

**Fig.1:**
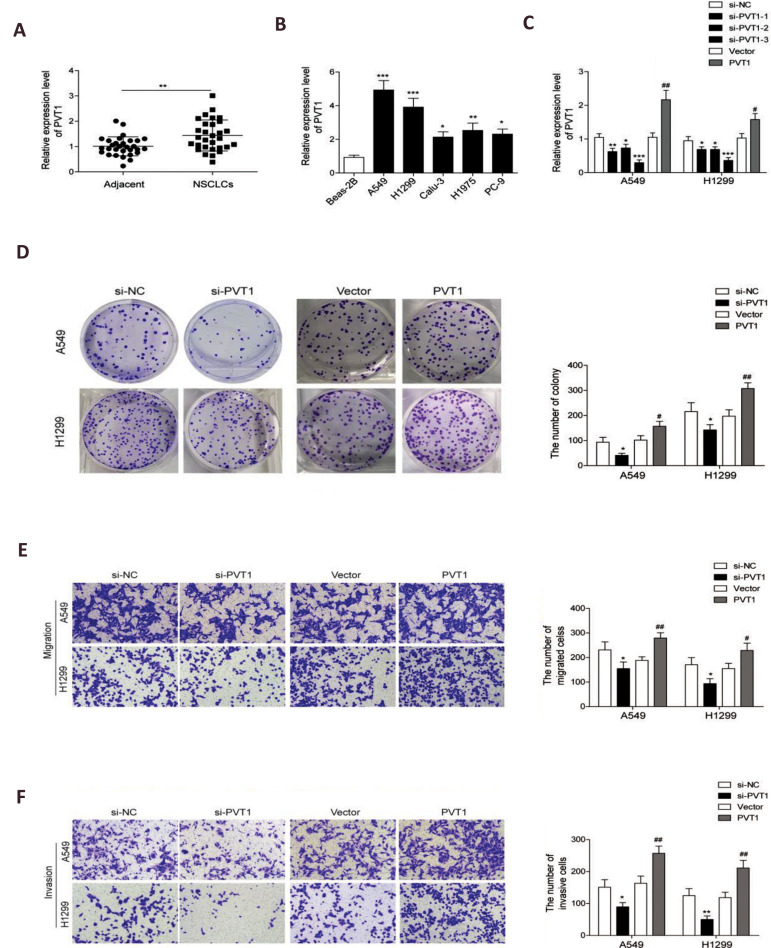
long non-coding RNAs (lncRNAs) plasmacytoma variant translocation 1(*PVT1*) was up-regulated in non-small cell lung cancer (NSCLC) tissues and cells, promoting NSCLC cell invasion and migration. **A. **quantitative reverse transcription polymerase chain reaction (qRT-PCR) analysis for the expression of PVT1 in the NSCLC and adjacent normal tissues (n=30). **B.** qRT-PCR analysis for the expression of PVT1 in five NSCLC cell lines compared to the normal lung epithelial cells, BEAS-2B. **C.** qRT-PCR analysis for the expression of PVT1 in A549 and H1299 cells transfected with three siRNAs of PVT1 (si-PVT1-1, siPVT1-2 and si-PVT1-3) or the negative control (si-NC) as well as pcDNA-PVT1 overexpression vector (PVT1) and pcDNA vector (vector). **D. **Colony formation assays were used to assess proliferation of A549 and H1299 cells after PVT1 knockdown and PVT1 overexpression. **E.** Matrigel-free transwell assay for cell migration and** F.** Matrigel transwell assay for cell invasion were confirmed in A549 and H1299 cells after PVT1 knockdown by siRNAs transfection and PVT1 overexpression. Data are mean ± SD of three independent experiments. *; P<0.05, **; P<0.01, ***; P<0.001 compared to si-NC group, #; P<0.05, and ##; P<0.01; P<0.01 compared to the vector group. *PVT1* knockdown inhibited expression of yes-associated protein 1 (YAP1) and NOTCH1 signaling activation.

To investigate the role of *PVT1* in NSCLC, we first analyzed expression pattern of this lncRNA in 30 NSCLC and adjacent normal tissues using qRT-PCR. Results showed that *PVT1* level in NSCLC tissues was significantly higher than the adjacent normal tissues (Fig .1A), indicating that *PVT1* may be involved in NSCLC progression. Similar results were obtained in NSCLC cell lines. *PVT1* expression in A549, H1299, Calu-3, H1975 and PC-9 cells was much higher than in BEAS-2B cells (Fig .1B). To examine whether *PVT1* was functionally involved in NSCLC, we selected NSCLC cells A549 and H1299 for further explorations. qRT-PCR assay was performed to determine efficiency of *PVT1* siRNA (applied for knockdown) and *PVT1* plasmid (used for overexpression). The results showed that *PVT1* expression was sufficiently downregulated after transfecting with the three siRNAs, and it was successfully upregulated after *PVT1* plasmaid transfection of both A549 and H1299 cells (Fig .1C). The most efficient si-PVT1-3 (collectively referred to as si-*PVT1* hereinafter) was selected for *PVT1* knockdown in the follow-up experiments. Then, colony formation assays were carried out to determine its effect on cell proliferation. It was determined that *PVT1* knockdown significantly inhibited cell proliferation, while *PVT1* overexpression significantly promoted cell proliferation (Fig .1D). Next, we evaluated the effects of *PVT1* on cell migration and invasion. As expected, the decreased expression of *PVT1* expression caused suppression of cell migration (Fig .1E) and invasion (Fig .1F), while *PVT1* overexpression promoted cell migration (Fig .1E) and invasion (Fig .1F). Taken together, these findings implied that lncRNA *PVT1* may function as an oncogene to promote tumorigenesis and development of NSCLC.

**Fig.2:**
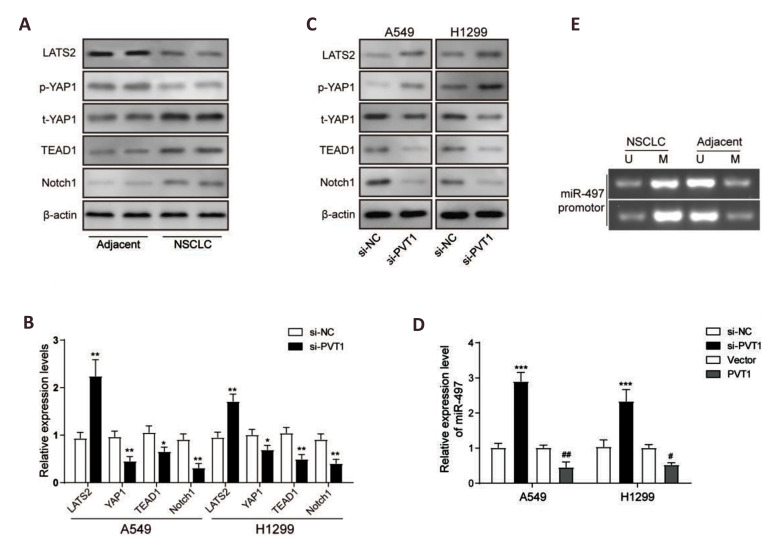
Plasmacytoma variant translocation 1(*PVT1*) knockdown inhibited yes-associated protein 1 (YAP1) expression and Notch1 signaling activation. Western blot analysis for protein levels of LAST2, p-YAP1, t-YAP1, TEAD and Notch1 in the NSCLC and adjacent normal tissues. **B.** Quantitative reverse transcription polymerase chain reaction (qRT-PCR) analysis for mRNA expression levels of *LAST2, YAP1, TEAD* and* Notch*1in A549 and H1299 cells after PVT1 knockdown using the treatment with si-PVT1 or si-NC. **C.** Western blot analysis for protein levels of LAST2, p-YAP1/t-YAP1, TEAD and Notch1 in A549 and H1299 cells after PVT1 knockdown by treatment with si-lncPVT1 or siRNA negative control (si-NC). **D.** qRT-PCR analysis for the expression of microRNA-497(miR-497) in A549 and H1299 cells after PVT1 knockdown or overexpression. **E.** MSP analysis for the methylation level of *miR-497* promoter. Data are mean ± SD of three independent experiments. *; P<0.05, **; P<0.01 compared to si-NC group;^ ##^; P<0.01 compared to vector group, p-YAP1; Phosphorylated YAP1 protein, t-YAP1; Total YAP1 protein, si-NC; siRNA negative control, si-PVT1; PVT1 siRNA for knockdown, Vector; Empty pcDNA vector for negative control of overexpression, A549 and H1299; Two of human lung carcinoma cell lines, MSP; Methylation-specific polymerase chain reaction, LAST2; Large tumor suppressor kinase 2 protein, and SD; Standard deviation.

Hippo/YAP and NOTCH signaling pathways are associated with the occurrence and development of NSCLC (21). Western blot analysis demonstrated that protein levels of LAST2 and relative phosphorylated YAP1 were significantly down-regulated in NSCLC tissues than that of the adjacent tissues, while total YAP1, TEAD and NOTCH1 proteins were up-regulated in NSCLC tissues than that of the adjacent tissues (Fig .2A), indicating that the Hippo pathway was suppressed, promoting NOTCH signaling pathway activation in NSCLC tumorigenesis. Furthermore, we further investigated the effects of lncRNA *PVT1* on those pathways by siRNA knockdown experiments. Results of qRT-PCR analysis showed that mRNA expression levels of *YAP1, TEAD1 *and *NOTCH1* were decreased obviously in A549 and H1299 cells after *PVT1* knockdown by siRNA treatment, except the mRNA level of *LAST2* which was up-regulated in si-*PVT1* group than the si-NC group in the A549 and H1299 cells (Fig .2B). Results of western blot analysis showed that LAST2 protein and phosphorylated YAP1 were significantly up-regulated while total YAP1, TEAD1 and NOTCH1 proteins were down-regulated after *PVT1* knockdown (Fig .2C). Taken together, our results demonstrated that *PVT1* knockdown could inhibit expression of YAP1 and NOTCH1 signaling activation.

Previous report found that knockdown of *PVT1* effectively promoted *miR-497* expression (13). To verify it, we detected *miR-497* expression levels after *PVT1* knockdown or overexpression in A549 and H1299 cells using qRT-PCR. Results showed that expression of *miR-497* was negatively regulated by *PVT1* (Fig .2D). To determine the role of lncRNA *PVT1* in regulating methylation, *miR-497* promoter methylation analysis was performed in the NSCLC and adjacent tissues by MSP. Results showed that methylation level of miR497 promoter was higher in the NSCLC tumor tissues than the adjacent tissues (Fig .2E), indicating that DNA methylation modification of *miR-497* promoter was occurred by tumorigenesis, which may be one reason for the low expression level of *miR-497* in NSCLC and it may also be one of results of lncRNA-mediated methylation.

### *PVT1* directly interacted with EZH2 in NSCLC cells

**Fig.3:**
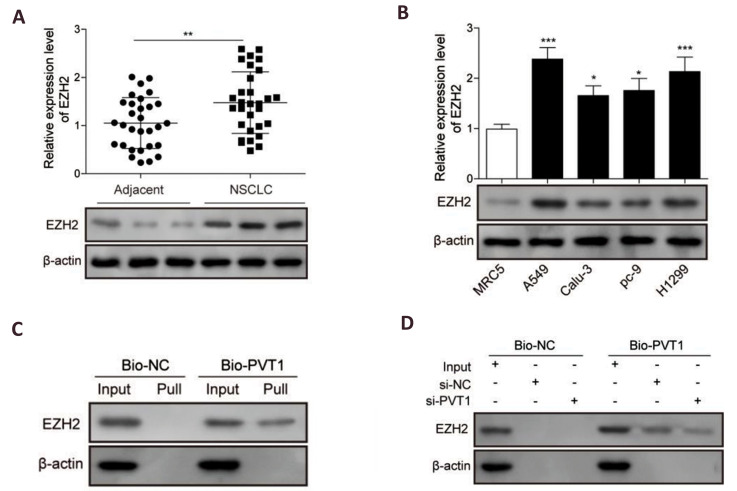
Long non-coding RNAs (lncRNAs)-plasmacytoma variant translocation 1(*PVT1*) directly interacted with enhancer of zeste homolog 2 (EZH2) in non-small cell lung cancer (NSCLC).** A.** Quantitative reverse transcription polymerase chain reaction (qRT-PCR) and western blot analysis, respectively for the mRNA and protein expression levels of EZH2 in the NSCLC and adjacent normal tissues (n=30). **B. **qRT-PCR and western blot analysis, respectively for the mRNA and protein expression levels of EZH2 in NSCLC cell lines compared to the normal lung epithelial cells, BEAS-2B. **C.** Relationship between lncRNA PVT1 and EZH2 verified in A549 cells by RNA pull-down and western blot assays using a biotin-labeled probe of PVT1 (bio-PVT1). **D. **RNA pull-down and western blot assays used to verify the PVT1 effect on EZH2 level after PVT1 knockdown by treating with si-lncPVT1 or si-NC in A549 cells. Data are mean ± SD of three independent experiments. *; P<0.05, **; P<0.01, ***; P<0.001 compared to the adjacent group or MRC5 group. si-NC; siRNA negative control, Bio-NC; Biotin-labeled negative control, Pull; Pull-down group, and SD; Standard deviation.

EZH2 is the functional enzymatic component of the polycomb repressive complex 2 (PRC2) and it has been linked to many forms of cancer. lncRNA *PVT1* was reported to modulate thyroid cancer cell proliferation by recruiting EZH2. To confirm whether *PVT1* could also interact with EZH2 in NSCLC, expression profile of EZH2 in clinic and cell levels were measured by qRT-PCR and western blot. The probable interaction between them was further verified using RNA pull-down assay. Results showed that EZH2 was significantly up-regulated in NSCLC tissues and cell lines compared to the normal one in both mRNA and protein levels (Fig .3A, B). RNA pull-down analysis identified the interaction between *PVT1* and EZH2 which was successfully pulled-down by a biotin-labeled *PVT1* probe in the A549 cell samples (Fig .3C). RNA pull-down experiments of the cell samples after transfecting with si-*PVT1* or siRNA-NC and culturing for 48 hours further proved these findings. Compared to the si-NC group, the level of EZH2 in the si*PVT1* group was reduced significantly along with *PVT1* knockdown (Fig .3D). All of these results have indicated that *PVT1* directly interacted with EZH2 in NSCLC.

### *PVT1* regulated the expression of YAP1 through EZH2-mediated *miR-497* promoter methylation

**Fig.4:**
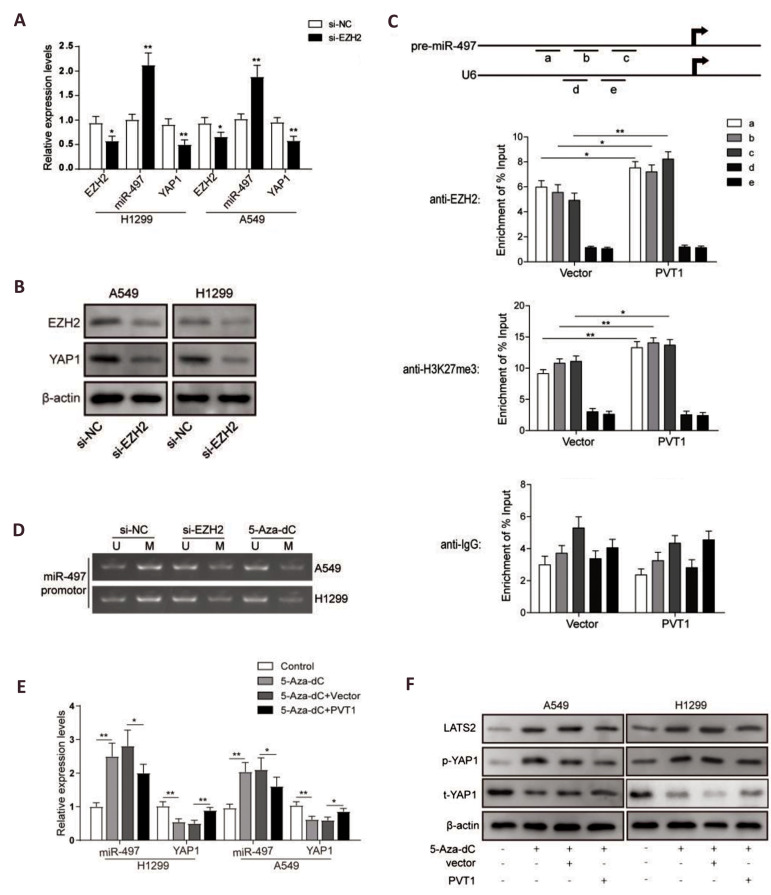
pcDNA-PVT1 vector for overexpression (PVT1) regulated expression of yes-associated protein 1 (YAP1) through enhancer of zeste homolog 2 (EZH2) -mediated microRNA-497(miR-497) promoter methylation. **A.** Quantitative reverse transcription polymerase chain reaction (qRT-PCR) analysis for the expression levels of *EZH2*, *miR-497* and *YAP1* in A549 and H1299 cells after EZH2 knockdown, treated with si-EZH2 or si-NC. **B.** Western blot analysis for the protein levels of EZH2 and YAP1 in A549 and H1299 cells after EZH2 knockdown, treated with EZH2 siRNA for knockdown (si-EZH2) or si-NC. **C. **ChIP–qPCR of EZH2 occupancy and H3K27me3 binding in the *miR-497* promoter in H1299 cells with or without PVT1 overexpression. a, b and c represented respectively three pairs of amplification primers for *miR-497* promoter; and d and e represented respectively two pairs of amplification primers for *U6* promoter as internal references. **D.** MSP analysis for the methylation level of *miR-497* promoter in A549 and H1299 cells treated with si-EZH2 or 5-Aza-dC. **E.** qRT-PCR analysis for the expression levels of *miR-497* and *YAP1* in A549 and H1299 cells treated with 5-Aza-dC or combined with PVT1 overexpression. **F.** Western blot analysis for the protein levels of LAST2 and YAP1 phosphorylation in A549 and H1299 cells treated with 5-Aza-dC or combined with PVT1 overexpression. Data are mean ± SD of three independent experiments, *; P<0.05, **; P<0.01 compared between siRNA negative control (si-NC) and si-EZH2 groups or compared between groups as shown with a horizontal line.5-aza2-deoxycytidine;A549 and H1299; Two of human lung carcinoma cell lines, ChIP; Chromatin immunoprecipitation, MSP; Methylation-specific polymerase chain reaction, LAST2; Large tumor suppressor kinase 2 protein, and SD; Standard deviation.

lncRNA *PVT1* was reported to recruit EZH2 into the promoter of target genes to induce methylation, therefore inhibiting transcription of the target genes. As *PVT1* could down-regulate LAST2 and up-regulate YAP1, we wondered whether it was involved in the regulation of methylation. First, we suppressed the expression of *EZH2* using siRNAs in A549 and H1299 cells to confirm relationship of *EZH2* and *YAP1* expression levels. As results shown in Figure 4A, the mRNA expression level of *EZH2* was successfully suppressed by si-*EZH2* in the both cell lines, while expression of *miR-497* was inversely increased after EZH2 knockdown, and YAP1, a known target gene of *miR-497*, was downregulated along with the upregulation of *miR-497* (Fig .4B). To verify the interaction between EZH2 with miR497 promotor, ChIP assays using EZH2 and H3K27me3 antibodies were performed. The qPCR results of *miR-497* promotor indicated that EZH2 could directly bind to the miR497 promoter region and mediate H3K27me3 modification, and the *PVT1* overexpression promoted these interactions (Fig .4C). Moreover, we detected methylation modification of the *miR-497* promoter with MSP after EZH2 knockdown or 5-Aza-dC treatment- an inhibitor commonly used for DNA methylation. Results showed that methylation level of the *miR-497* promoter was significantly decreased in A549 and H1299 cells in si-*EZH2* and 5-Aza-dC groups compared to the control group (Fig .4D), further suggesting that expression of the *miR-497* may be regulated by EZH2 through DNA methylation of the corresponding microRNA promoter. Besides, after treatment with DNA methylation inhibitor 5-Aza-dC, expression of *miR-497* was increased obviously. This could be inversed by combined treatment with *PVT1* overexpression, while the changes of YAP1 expression were on the contrary (Fig .4E). As expected, the protein levels of LAST2 and phosphorylated YAP1 were significantly upregulated after 5-Aza-dC treatment, which could be inversed by *PVT1* overexpression (Fig .4F). Taken together, these results indicated that *PVT1* regulated expression of *miR-497* and YAP1 through EZH2/H3K27me3-mediated *miR-497* promoter methylation modification.

### *PVT1* promoted NSCLC cells epithelial-mesenchymal transition and migration through activation of NOTCH1 signaling pathway

**Fig.5:**
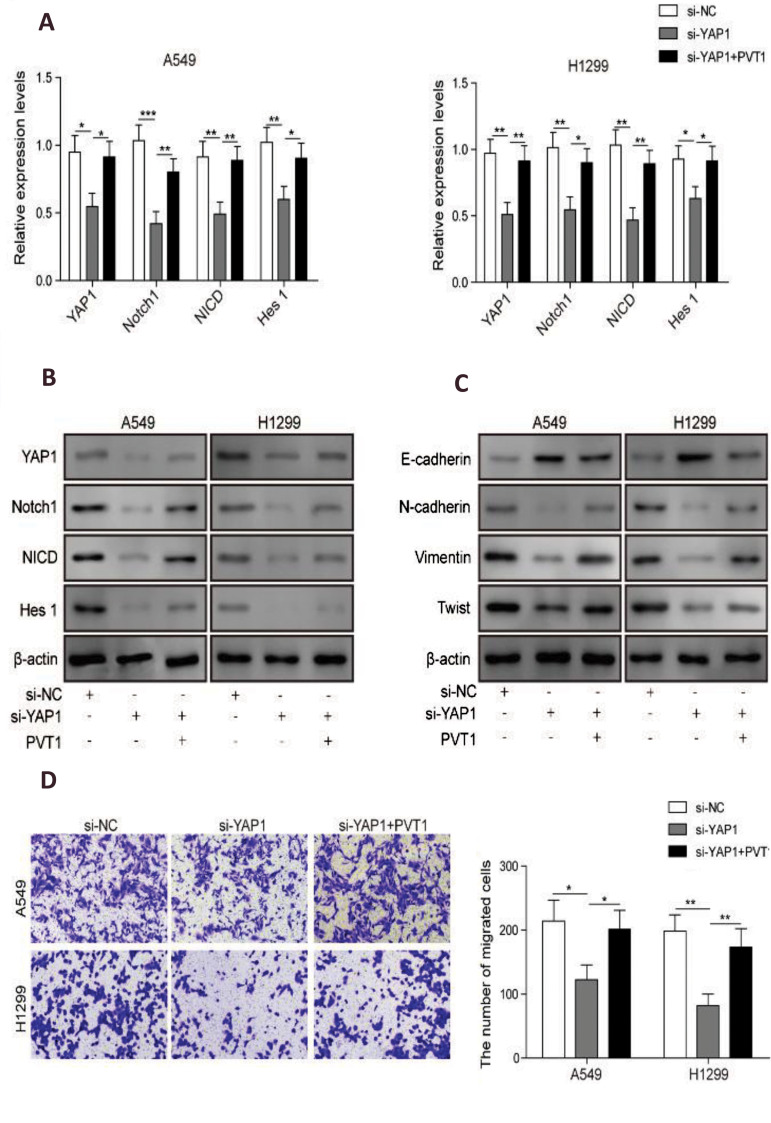
Long non-coding RNAs (lncRNAs)-plasmacytoma variant translocation *1(PVT1)* promoted invasion and epithelial-mesenchymal transition (EMT) of nonsmall cell lung cancer (NSCLC) cells through activation of notch receptor 1 protein (Notch1) signaling via yes-associated protein 1 (YAP1). **A. **Quantitative reverse transcription polymerase chain reaction (qRT-PCR) analysis for the expression levels of the key protein molecules in Notch signaling pathway of A549 and H1299 cells, after YAP1 knockdown or co-transfection with PVT1 overexpression. **B.** Western blot analysis for the key proteins in Notch signaling pathway and **C. **marker proteins of EMT in A549 and H1299 cells after YAP1 knockdown or co-transfection with PVT1 overexpression. **D. **Matrigel-free transwell assay for cell migration were confirmed in A549 and H1299 cells, after YAP1 knockdown or co-transfection with PVT1 overexpression. Data are mean ± SD of three independent experiments, *; P<0.05, **; P<0.01, ***; P<0.001 compared between groups as shown with a horizontal line, si-NC; siRNA negative control, si-YAP1; YAP1 siRNA for knockdown, A549 and H1299; The human lung carcinoma cell lines, and SD; Standard deviation.

To test whether *PVT1* regulates invasion of NSCLC cells through activation of NOTCH1 signaling and epithelial-mesenchymal transition (EMT), expression of YAP1, NOTCH1, NICD and HES1 were assessed in A549 and H1299 cells after YAP1 knockdown with or without *PVT1* overexpression co-transfection. Results showed that YAP1 knockdown suppressed expressions of NOTCH1, NICD and HES1 (Fig .5A, B), indicating that activation of Hippo signaling pathway suppressed activation of NOTCH signaling pathway; while *PVT1* overexpression compensated these effects (Fig .5A, B), indicating that *PVT1* promoted activation of NOTCH signaling pathway by suppressing Hippo signaling pathway through YAP1 overexpression. EMT markers (N-CADHERIN, VIMENTIN, E-CADHERIN and TWIST1) were downregulated in A549 and H1299 cells, while *PVT1* overexpression reversed these effects, indicating that *PVT1* promoted EMT of NSCLC cells through upregulated YAP1 (Fig .5C). Then, we further evaluated their effects on cell migration. Transwell assays demonstrated that YAP1 knockdown could significantly diminish migration and invasion abilities of A549 and H1299 cells. These effects were reversed by *PVT1* overexpression (Fig .5D). Taken together, our results demonstrated that lncRNA-*PVT1* promoted invasion and EMT of NSCLC cells through activation of NOTCH1 signaling.

## Discussion

NSCLC accounts for almost 80% of lung cancer, as the leading cause of cancer mortality (22). Even though great progresses have been made in surgical resection, chemoradiotherapy or target drugs, its prognosis is still poor (2). Hence, it is of most importance to uncover the molecular mechanism of carcinogenesis. Accumulating studies have shown that some lncRNAs associate with NSCLC generation and they participate in different biological processes in NSCLC (23). Previously, *PVT1* was reported to function as an oncogenic lncRNA, a potential prognostic biomarker and therapeutic target in NSCLC (23-24). It was reported to act as a competing endogenous RNA (ceRNA) for *miR-497* in promoting NSCLC progression (13)and it could regulate expression of HIF1α via functioning as ceRNA for* miR-199a* in NSCLC (25). It also facilitates NSCLC cell invasion through MMP9 (26). Besides, *PVT1* could promote NSCLC cell proliferation through epigenetically regulating LATS2 expression (9). Here, consistent with previous study, we confirmed that *PVT1* was upregulated in NSCLC tissues and cells and it might promote proliferation and migration in NSCLC, indicating that *PVT1* may serve as an oncogene to promote NSCLC development and progression.

Hippo pathway is involved in the development of NSCLC. LATS1, the core component of Hippo pathway, was reported to suppress NSCLC cell proliferation and migration (27). While, Tafazzin (TAZ) was reported to be overexpressed in 70% NSCLC cell lines and it could cause transformation of non-tumorigenic lung epithelial cells (28). Besides, constitutively activated YAP, a TAZ paralog, was reported to drive NSCLC progression and metastasis (29). In this study, we found that *PVT1* regulated expression of YAP1 through EZH2-mediated *miR-497* promoter methylation. These findings suggest that *PVT1* regulated Hippo-NOTCH1 signaling pathway through epigenetic regulation. Besides, *PVT1* activated NOTCH1 signaling through YAP1, indicating that YAP1 is most likely a NOTCH1 upstream driver gene and we, for the first time, revealed the molecular mechanism of NOTCH1 upregulation in NSCLC.

Increasing evidences showed that EZH2 contributed to malignant transformation (30). *PVT1* and EZH2 were also reported to have correlations. For example, *PVT1* modulates cell proliferation and apoptosis by recruiting EZH2 in HCC (11). While, it represses ANGPTL4 transcription through binding with EZH2 in trophoblast cell (31). Besides, it can promote cell proliferation and invasion via targeting EZH2 in glioma cells (32). In this study, RNA pull-down and RNA binding protein immunoprecipitation (RIP) assays were performed to verify the relationship between *PVT1* and EZH2. They demonstrated that *PVT1* could recruit EZH2 to the promoter of *miR-497*, thus mediate H3K27me3 modification. We are the first to clearly elucidate the molecular mechanism by which *PVT1* inhibits *miR-497* in NSCLC.

EMT progression is of great importance for migration of NSCLC cells (33). Investigations on EMT will be of great benefit to the bulk of solid malignant tumors, as most of human malignancies arise from the epithelium tissues 33. However, the functional role of lncRNA in modulating EMT in NSCLC is still poorly understood. Here, we identified *PVT1* as a novel player in modulating EMT progress and we also revealed a previously unknown mechanism. Regarding that microRNAs may function as oncogenes or tumor suppressors in almost all kind of tumors, including NSCLC (34), identifying and exploring their functions may do us a great favor to develop more effective biomarkers and therapies. Previously, *miR-497* was reported to suppress TGF-β induced EMT of NSCLC by targeting METADHERIN (MTDH) (35). Here, consistent with previous reports, we uncovered that miR497 inhibits EMT of NSCLC cells via targeting YAP1. 

## Conclusion

In summary, we demonstrated that *PVT1* is an oncogene in NSCLC, overexpression of which could promote cell proliferation, migration and EMT process via regulating the expression of YAP1 through EZH2-mediated *miR-497* promoter methylation. This novel mechanism will lead to a better understanding of EMT in NSCLC or even other cancers. Overall, these findings indicate that *PVT1* might be a potential target for NSCLC therapy.
